# Tugs-of-war in science

**DOI:** 10.1172/JCI165312

**Published:** 2022-10-17

**Authors:** Mitchell A. Lazar

**Affiliations:** 1 Institute for Diabetes, Obesity, and Metabolism, Perelman School of Medicine at the University of Pennsylvania, Philadelphia, Pennsylvania, USA.; 2 Division of Endocrinology, Diabetes, and Metabolism, Department of Medicine, University of Pennsylvania Perelman School of Medicine, Philadelphia, Pennsylvania, USA.

One of the greatest honors of my career was receiving the 2009 Stanley J. Korsmeyer Award from the American Society for Clinical Investigation. Stan Korsmeyer was a great physician-scientist who was lost too soon, at the age of 54, but not before he’d made transformational discoveries that revolutionized thinking about oncogenes and programmed cell death. Among Stan Korsmeyer’s major discoveries were BCL-2 (1), which acts in mitochondria to prevent apoptosis (2), and the related BAX, which antagonizes the actions of BCL-2 and promotes cell death (3). Korsmeyer used the metaphor of a rheostat to describe how the ratio of BCL-2 to BAX determines how cells respond to an apoptotic stimulus, in a tug-of-war over the life and death of a cell (4).

Scientific tugs-of-war can occur not only between molecules but between scientists. The reasons can vary from differing interpretations of data to mutually exclusive findings. My own path to the Korsmeyer award involved tugs-of-war between molecules as well as scientists and began in the late 1980s, when I was performing research as an endocrinology fellow in the lab of Bill Chin at Brigham and Women’s Hospital. This was the golden era of molecular endocrinology, and when I joined the lab the first nuclear hormone receptors had recently been molecularly identified. I was particularly interested in thyroid hormone (TH) action, and after similar but distinct TH receptors (TRs) were described in chickens (5) and humans (6), I decided to investigate the TR heterogeneity further by screening my own newly minted cDNA library from a highly TH-responsive rat pituitary cell line with a hybridization probe containing DNA sequences complementary to human TRβ.

This was an exciting time, when protein-coding genes were just being identified and characterized, but the process was painstakingly slow and prone to frustration, as the techniques for DNA sequencing were primitive in comparison with today’s next-generation sequencing tools. I characterized a number of rat cDNAs related to the human TR and, as Rich Hodin and I marched through the sequencing 100 base pairs at a time, I was thrilled by the recognition that two clones were different from any of the TRs that had been reported. One of the new isoforms was most related to the original human TR (now known as TRβ1) and turned out to be an unexpected pituitary-selective TR that we termed TRβ2 (7, 8) (Figure 1A).

The other new isoform was more similar to the TR that had been identified in chickens (now known as TRα), but it possessed a C-terminus that was distinct from all other TRs. Currently known as TRα2, we called this molecule c-erbAα2 since TRα was the cellular homolog of the erbA oncogene (Figure 1A). As we were getting ready to publish this new TR, the only thing remaining was to show that it bound TH. This should have been quite straightforward, since TH contains iodine and could be radiolabeled to high specific activity, a key component of ligand binding assays. However, unlike the other TRs that we could easily demonstrate to bind labeled triiodothyronine (T3, the active form of TH), we could not discern binding of T3 to TRα2 above the background (negative control) of the assay (Figure 1B).

While we were repeating and optimizing our TH binding experiments, a paper in the November 6, 1987 issue of *Science* magazine described a new human form of TRα that was clearly the homolog of our rat TRα2. The results were remarkably similar to ours, with one glaring difference; human TRα2 was reported to bind TH with high affinity and specificity (9). We tried again and again with our rat TRα2, but no matter how many times we performed the assay, we found no evidence that it could bind TH, even though positive controls using other TRs worked just fine. In the meantime, the April, 1988 issue of *PNAS* included a paper by a different group describing human TRα2 as a bona fide and functional TR (10).

As a physician-scientist in training with aspirations to run my own independent laboratory, I was despondent. Negative results are always harder to publish than positive ones, and the perceived impact is much less. However, we had confidence in our own data, and we described our cloning of TRα2 and its inability to bind TH in the October, 1988 issue of *Molecular Endocrinology* (11). Reassuringly, Izumo and Mahdavi independently and nearly simultaneously reported findings similar to ours, i.e., they too cloned the rat TRα2 isoform but found no evidence that it could bind TH (12). Moreover, the possibility of species-specific functions was excluded by direct comparison of the human and rat TRα2 proteins (13).

Hence the scientific tug-of-war: four groups identified the novel TRα2 isoform around the same time, all agreed that the TRα2 isoform was generated by alternative splicing of the 3′ end (C-terminus) of the TRα gene, but there was a fundamental difference in the conclusions about its function. Two found that TRα2 bound TH, while two had clear evidence against this. What was going on, and how could this be resolved? To address this, we teamed up with Ron Koenig, Greg Brent, Reed Larsen, and David Moore, who had an assay for TR-regulated transcription up and running. We reasoned that if TRα2 could not bind TH, then it would be likely to act as a dominant inhibitor of TRα1 since TRα1 and TRα2 had identical DNA binding regions that should recognize the same target genes. In a wonderful collaboration, published in the February 16, 1989 issue of *Nature*, we showed that this was indeed the case; TRα2 was in a functional tug-of-war with TRα1, and the transcriptional outcome was determined by the ratio of TRα1 to TRα2 (14). This was a new concept in transcriptional regulation, and gratifyingly, a number of additional examples of a single gene encoding functionally competitive activators and repressors were discovered over the next few years (15) (Figure 1C).

The demonstration of the molecular tug-of-war between TRα1 and TRα2 apparently had an impact on the scientific tug-of-war as, to my knowledge, no additional papers claimed that TRα2 could bind TH after the publication of the dominant negative activity of TRα2. By contrast, although the biological function of TRα2 is still not well understood, numerous papers have subsequently confirmed its inability to bind TH. Indeed, as more is now known about the structure and function of the ligand-binding C-terminus of nuclear receptors, it is clear that the inability of TRα2 to bind TH should not be not surprising because its unique C-terminus lacks critical features important for ligand binding as well as transcriptional activation and repression by other nuclear hormone receptors. As of this writing, the “TRα2 is not a bona fide TH receptor” side appears to have won the tug-of-war (16).

What is to be learned from this? Readers are encouraged to judge for themselves, but I will suggest a few takeaway lessons. First, a specific scientific finding published in high impact peer-reviewed journals may not be correct even if it is described by two seemingly independent groups. If conflicting studies are performed well, with all possible controls, then they should be taken seriously, even if the results are negative. Although it is often not clear why some positive results are not reproducible, possibilities include that the groups were trying to “prove” rather than “test” their hypothesis (e.g., that TRα2 binds TH) and there could be unconscious bias, especially if the positive result seemed logical (e.g., TRα2 is so highly related to other TRs). Intense competition to be the first to publish may also contribute to irreproducible results.

For those struggling to make sense of a negative result, it is imperative to make sure that the experiment is done correctly, multiple times, and with all negative and positive controls. If, after that, the results contradict a published finding, then it is wise to use this knowledge to discover something new rather than focusing on policing the field. Presenting the findings as a new paradigm rather than a correction can enhance the impact of the work. I have encountered a number of similar situations in my scientific career, and had variable success convincing reviewers and editors of the impact of the negative finding even when the result that it contradicts is potentially misleading and widely cited.

Tugs-of-war between scientists are critical to the scientific enterprise. We aspire to the truth, and to the extent that our hypotheses are supported by data the scientific method brings us closer to it. However, no matter how much evidence in favor of a hypothesis, there is always the chance that it will be disproved by future experiments. New or questionable hypotheses should be particularly vulnerable. On the other hand, per Bayes’ theorem, the bar should be much higher to overturn longstanding hypotheses backed by lots of solid data. At the molecular level, tugs-of-war between molecules are examples of hypotheses proposed to explain biological observations. Stan Korsmeyer’s hypothesis that the tug-of-war between BCL-2 and BAX controls whether cells live or die has held up to experimental scrutiny and been foundational for the development of a highly effective class of cancer therapeutics, long after his untimely passing. This is one of the greatest legacies that a scientist can hope for.

## Figures and Tables

**Figure 1:**
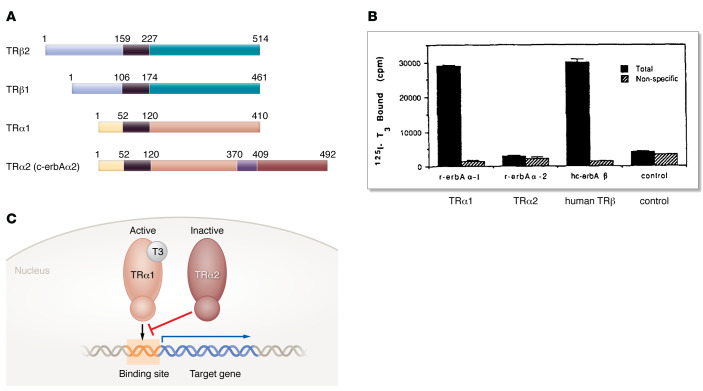
Thyroid hormone receptor α2. (**A**) Multiple forms of thyroid hormone receptors (TRs). Amino acid numbers refer to those in the rat proteins. Adapted with permission from Lazar (8). (**B**) Data from Lazar et al. (11) demonstrating that TRα2 does not bind thyroid hormone. Rat TRα2 (r-erbAα-2) produced in rabbit reticulocyte lysate does not bind thyroid hormone (T3). Positive controls for TRα1 (r-erbAα-1) and human TRβ (hc-erbA β) with robust T3 binding are shown. Data reproduced with permission from Lazar et al. (11) and relabeled for nomenclature consistency. (**C**) Tug-of-war between thyroid hormone–binding, transcriptionally active TRα1 and inactive TRα2 competing for binding sites in target genes. cpm, counts per minute.

## Version 1 10/17/2022

Electronic publication
